# Experience with Apis—A Clinical Note

**Published:** 1887-04

**Authors:** Wm. A. Hawley

**Affiliations:** Syracuse, N. Y.


### EXPERIENCE WITH APIS.—A CLINICAL NOTE.

WM. A. HAWLEY, M. D., SYRACUSE, N. Y.

TO THE HOMŒOPATHIC PHYSICIAN.

MESSRS. EDITORS:—I had a letter three days ago which interested me so much that I transcribe it. If you think it of sufficient value you may print it in your journal. I will only premise that the patient, aged about fifty-eight years, is suffering with epithelioma of the uterus, and has been under my care and observation for the past three years, during which the growth has made no progress, but has rather receded, while her general health has greatly improved. On the first of September, 1886, she used, for the stinging, burning pains in the local trouble, a *single* dose of Apis.$^{cm}$, Fincke. Two days ago she wrote me that the stinging and burning had returned. I sent a single dose of Apis.$^{52m}$, Fincke, which she took as told in the letter. The result interests *me*.

DANBURY, February 23d, 1887.

MY DEAR DOCTOR:

I took those powders as directed, and must tell you of a singular experience I had Saturday night or Sunday morning. As usual, Nathan (her husband) was late home, and it was nearly twelve before we went to bed, although I had been lying down a good deal of the time before he came. I took a powder about eleven o'clock; did not get to sleep in some time, and after turning in bed felt something wrong about my forehead, and thought my hair had got twisted so as to pull pretty sharp. I brushed it two or three times with my hand, but it kept pulling worse, and I thought I felt something on the pillow, and imagined it might be a wasp or spider. I gave a shriek that aroused Nathan, and asked him to light a lamp, something had stung me. He did so, and we searched the bed, but could find nothing. I soon felt another sting on my left leg, two or three inches below the hip joint. I made sure there was nothing there, but two bunches came up like two small beans, and there was also a bunch on my forehead. They appeared in every way like a bee sting. I put some Arnica on my head, but after the one on my leg came I began to think about the medicine you were giving me for stinging pains, and did not make any further application. Was it the medicine? I have not had any of the stinging pain from the local trouble worth mentioning since, but I have had some pretty hard piercing pain the past week, and also burning, so as to make me think there is a good deal of trouble there yet. * * * Yours, sincerely,

J. S. G.

I must add that this patient has never had any idea what remedy she was taking. Of course, she knows nothing of what the drug was in this case.

